# Amide proton transfer weighted contrast has diagnostic capacity in detecting diabetic foot: an MRI-based case–control study

**DOI:** 10.3389/fendo.2024.1287930

**Published:** 2024-03-21

**Authors:** Shan Lu, Jiwei Tian, Shiyu Zhao, Xueyan Song, Xianglu Meng, Guangyang Ma, Dengping Liu, Zhiwei Shen, Baocheng Chang

**Affiliations:** ^1^ NHC Key Laboratory of Hormones and Development, Tianjin Key Laboratory of Metabolic Diseases, Chu Hsien-I Memorial Hospital and Tianjin Institute of Endocrinology, Department of Radiology, Tianjin Medical University, Tianjin, China; ^2^ Clinical Science, Philips Healthcare, Beijing, China

**Keywords:** diabetic foot, ASL, APTw, infection, diabetes mellitus

## Abstract

**Objective:**

To evaluate the role of foot muscle amide proton transfer weighted (APTw) contrast and tissue rest perfusion in quantifying diabetic foot (DF) infection and its correlation with blood parameters.

**Materials and methods:**

With approval from an ethical review board, this study included 40 diabetes mellitus (DM) patients with DF and 31 DM patients without DF or other lower extremity arterial disease. All subjects underwent MRI, which included foot sagittal APTw and coronal arterial spin labeling (ASL) imaging. The normalized MTRasym (3.5 ppm) and the ratio of blood flow (rBF) in rest status of the affected side lesions to the non-affected contralateral side were determined. The inter-group differences of these variables were evaluated. Furthermore, the association between normalized MTRasym (3.5 ppm), rBF, and blood parameters [fasting blood glucose (FBG), glycosylated hemoglobin content, C-reactive protein, neutrophil percentage, and white blood cell count] was explored. Using an ROC curve, the diagnostic capacity of normalized MTRasym (3.5 ppm), BF, and blood biochemical markers in differentiating with or without DF in DM was assessed.

**Results:**

In the DF group, MTRasym (3.5 ppm) and BF in lesion and normalized MTRasym (3.5 ppm) were higher than those in the control group (p < 0.05). In addition, correlations were identified between normalized MTRasym (3.5 ppm) and blood parameters, such as C-reactive protein, glycosylated hemoglobin content, FBG, neutrophil ratio, and white blood cell (p < 0.001). Meanwhile, association between BF in lesion and blood parameters, such as C-reactive protein, neutrophil percentage, and FBG (p < 0.01). AUC of normalized MTRasym (3.5 ppm) in identifying with/without DF in patients with DM is 0.986 (95% CI, 0.918–1.00) with the sensitivity of 97.22% and the specificity of 100%.

**Conclusion:**

Normalized MTRasym (3.5 ppm) and the BF in lesion may be treated as a safer and more convenient new indicator to evaluate the tissue infection without using a contrast agent, which may be useful in monitoring and preoperatively assessing DF patients with renal insufficiency.

## Introduction

Diabetic foot (DF), as one of the complications of diabetic mellitus (DM), is of growing concern. Approximately 40 million individuals in China and 30 million people in the United States have DM, and there is a lifetime risk of 25% in China and 20% in the United States for developing DF, which has extended curative cycles, high disability rates, and higher mortality rates (1, 2). Local infection is the main cause of diabetic foot ulcers (DFUs) and eventual amputation. Bacterial growth within DFUs is facilitated by a number of variables, including poor glycemic control, immunosuppression, peripheral vasculopathy, and peripheral neuropathy. Wagner classification is commonly used in evaluating the prognosis of DF and achieving the optimal treatment for the patient, but it is mainly based on subjective assessment. Accurate quantitative assessment of information on infection and blood perfusion in the local tissues are essential for the therapeutic management of DF.

MRI is the effective method to assess soft-tissue infections and osteomyelitis in the foot with the presence of hyperintensity in fat-suppressed T2-weighted imaging or short time of inversion recovery (STIR) imaging (3). Meanwhile, dynamic contrast enhancement (DCE), diffusion-weighted imaging (DWI), and Dixon-based fat suppression have been used for detecting neuropathic arthropathy and osteomyelitis (OM) with more sensitivity and specificity. These MR techniques, however, do not quantify data about tissue molecular changes (1). Although computed tomography perfusion imaging (CTP) (4) and dynamic contrast-enhanced (DCE) magnetic resonance perfusion-weighted imaging (PWI) are used for evaluating the degree of tissue ischemia and the status of micro-vessels, the injection of high-dose contrast agents for CTP and PWI poses a significant risk to the patients, which may terminate in renal failure. Serum biomarkers such as C-reactive protein and leukocyte count could be used to differentiate necrotizing soft-tissue infections from non-necrotizing soft-tissue infections, but their diagnostic performance is still being debated (5). Therefore, a non-invasive risk-free imaging approach to quantify the molecular information and the blood flow in DF lesions and surrounding muscle issue is demanded.

Amide protons transfer weighted (APTw) imaging is a molecular MRI technique that uses an endogenous contrast agent, a saturation pulse at a specific frequency of the amide content of peptides and tissues protein, to achieve a magnetic transfer ratio of amide proton exchanged to free water proton (6). APTw signals were measured using magnetization transfer ratio asymmetry at 3.5 ppm [MTRasym (3.5 ppm)] to reflect the protein-weighted signal changes in glioma and musculoskeletal diseases (7). Zhang Hong et al. reported that hyperintense on the APTw images was observed in brain abscesses, viral encephalitis, and meningitis (8). Debnath et al. also reported that a higher mean APT-weighted contrast was found in brain-infective mass lesions (9). Furthermore, the majority of necrotizing soft-tissue infections are polymicrobial infections. BacCEST MRI makes it possible to identify, classify, and keep track of bacterial infections in rat brain with a bacterial abscess (10). CryptoCEST contrast enabled the detection of cryptococcomas in the brains of mice (11). The necrotic tissue and interstitial exudate from the DF lesions are rich in protein. Therefore, we hypothesis that MTRasym (3.5 ppm) in the DF lesions could be detected to evaluate the degree of inflammation in DF. Normalized MTRasym (3.5 ppm) [nMTRasym (3.5 ppm)] was calculated as the ratio of the APTw signal at the lesion to the surrounding muscular tissue to reducing the impact of multiple factors on APT outcomes, including tissue T1, irradiation strength, B1 inhomogeneity perturbing the magnetization, and magnetization transfer (MT).

Arterial spin labeling (ASL) MRI provides the cerebral blood flow (CBF) to quantify the perfusion of local tissues using magnetically labeled arterial blood as an endogenous contrast agent. The CBF was calculated from the difference between the labeled blood water and the unlabeled blood water with correcting for T1 relaxation time, blood-tissue partition coefficient, and transit time of the blood water to tissue water. The reduced CBF was found in patients with type 2 diabetes mellitus (T2DM) and patients with hypoglycemia (12, 13). Besides the CBF, the BF dynamics in skeletal muscle (14) and foot ulcers (15, 16) were also evaluated in diabetes mellitus patients at rest and with physical activity using ASL imaging. However, the results were inconsistent. Edalati et al. observed that peri-ulcer exercise perfusion was lower than away-ulcer exercise perfusion, while Pantoja et al. found that peri-wound tissue perfusion was increased relative to the rest of the foot. It may be more valuable to explore the differences in local perfusion in diabetic foot patients at rest with a larger sample size in clinical practice. Moreover, the ratio of BF (rBF) in the diseased area of the afflicted foot to the same area of the contralateral foot may decrease the individual variances and increase the strength of the results.

This study aimed to explore the use of foot APTw in combination with ASL imaging to evaluate the infection and blood perfusion in DF and investigate the relationship among rBF, nMTRasym (3.5 ppm), and blood biochemical parameters, such as FBG, glycosylated hemoglobin, neutrophils, and white blood cells. This diagnostic tool may be a reliable non-invasive means of evaluating the diabetic foot, especially for patients with kidney injury.

## Materials and methods

### Participants

**Figure 1:**
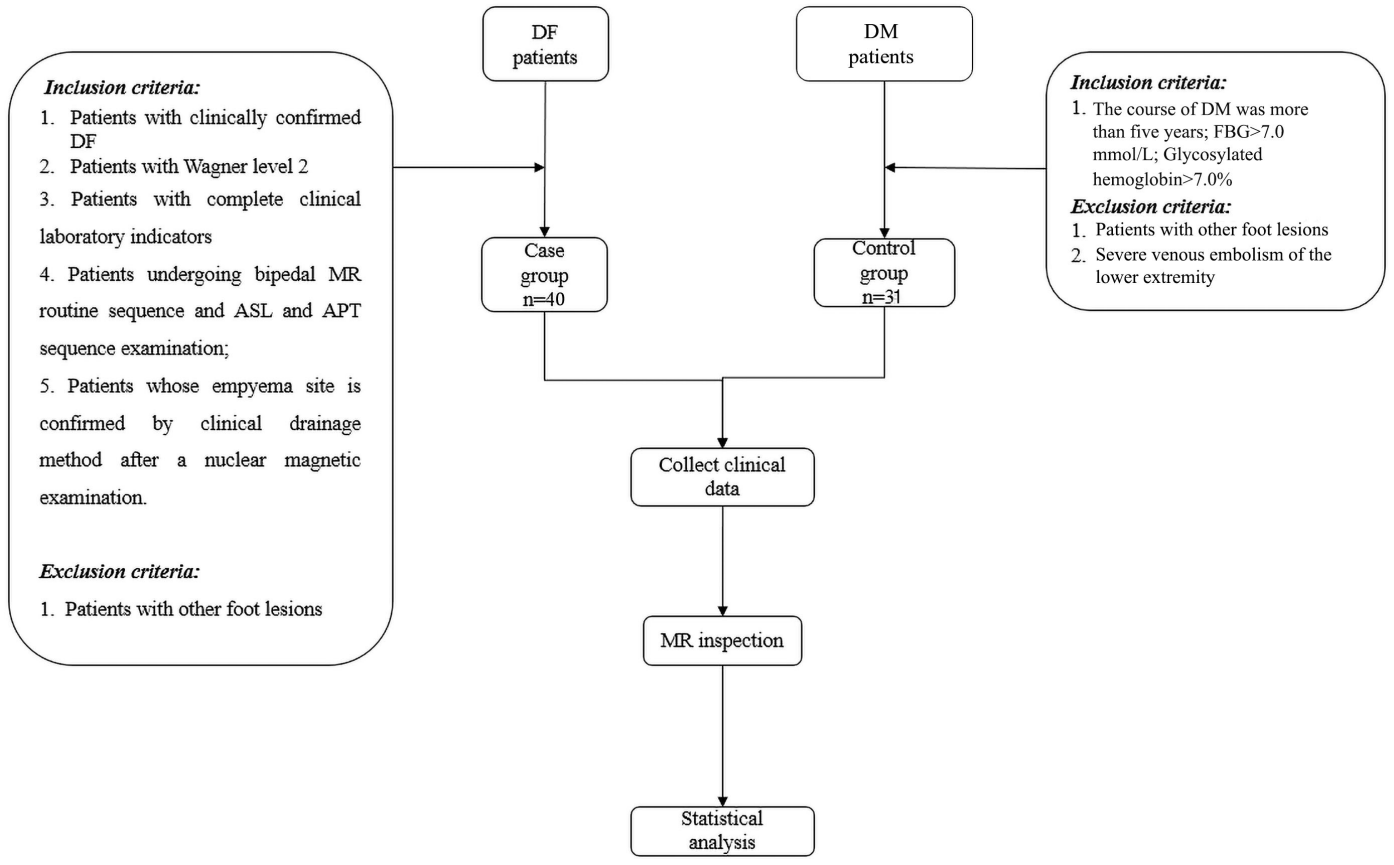
Flow chart of the study design.

Upon the approval of the study protocol by the ethics committee of our hospital (approval number DXBYYkMEC2021-09), 40 DM patients (30 male and 10 female patients, with an average age of 61.38 ± 13.20 years) diagnosed with DF from December 2020 to September 2021 in our hospital were assigned to the DF group. A total of 31 DM patients (18 male and 13 female patients, with an average age of 59.71 ± 11.76 years) without DF and lower extremity arterial disease were assigned to the control group. The inclusion criteria for the DF group were as follows: 1. patients with clinically confirmed DF; 2. patients with grade 2 according to the commonly used Wagner Grading Criteria (WGC) for DF (2); 3. patients with complete clinical laboratory evaluations; 4. patients who underwent routine MRI and ASL and APT sequence examinations; and 5. patients with confirmed soft tissue abscess by draining after MR examination. The inclusion criteria for the control group were as follows: 1. the course of DM was more than 5 years and 2. FBG >7.0 and glycosylated hemoglobin > 7%. Patients with other foot lesions or with severe venous embolism of the lower extremity were excluded. The flow chart of the study design is shown in **Figure 1**. Data on age and gender, fasting blood glucose (FBG) level, glycosylated hemoglobin content, white blood cell count, and neutrophil percentage were also collected.

### MR examination

All participants underwent foot MR examination using a 3.0-T MR scanner (Philips Ingenia CX, Best, Netherlands). After the patient lay supine with the feet advanced, two dS flex coils were placed in parallel and fixed to both sides of the feet. First, the conventional sagittal and axial T1-weighted, sagittal T2-weighted, coronal PD-weighted imaging with fat suppression, and coronal DWI imaging were performed. Then, axial ASL imaging based on the FFE-EPI sequence was acquired at post-label delay (PLD) times of 1,600 ms. Finally, a sagittal APTw sequence based on a 3D-modified Dixon-TSE sequence was performed with a saturation power of 1 µt and a duration of 1.8 s. B0 mapping was automatically calculated and was used to auto-correct the B0 magnetic field inhomogeneity of APTw images on the MRI console. The detailed sequence parameters are shown in **Table 1**.

**Table 1:** MRI sequence parameters.

	FOV (mm^2^)	Matrix	Thickness (mm)	Slice Gap (mm)	NSA	TSE factor	SENSE factor
T1WI	180270	256360	3.5	0.35	1	2	1.5
PDWI	180270	328340	3.5	0.35	1	10	3.0
T2WI	160130	210126	3.5	0.35	2	12	2.0
ASL	380320	180160	6.0	1.0	1	1	2.0
DWI	255180	128100	3.5	0.35	2	1	1.5
APT	175120	6443	6.0	0.0	1	170	1

### Post-processing and measurement

After MR examination, all images were transferred onto a workstation (IntelliSpace Portal, Version 9.0, Philips Healthcare) for data post-processing, and BF mapping and APTw mapping were automatically calculated. The data in the region of interest (ROI) was measured by two radiologists with extensive diagnostic experience (> 5 years) (17).

**Figure 2:**
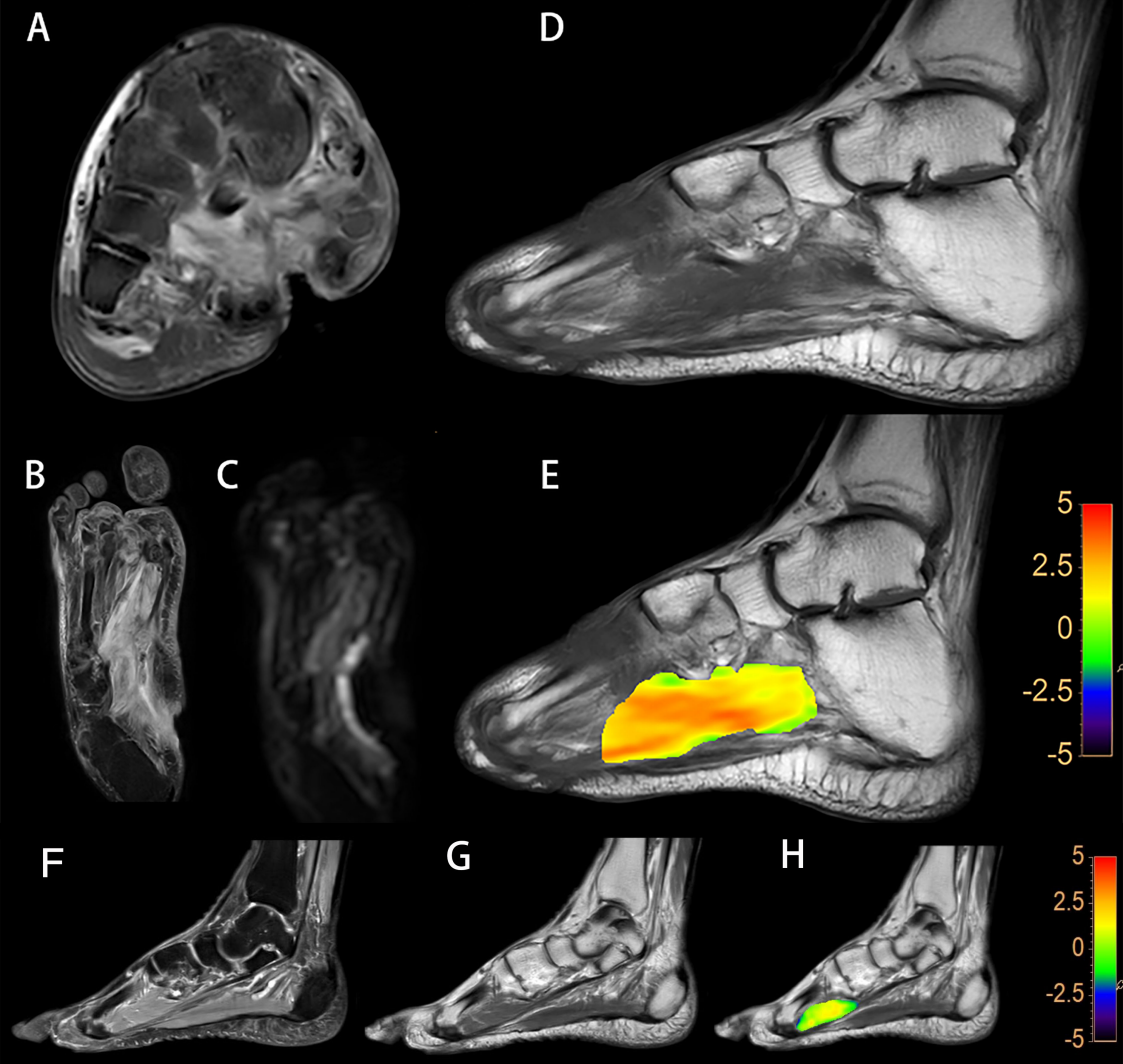
A diabetic foot of DM patient **(A–E)**. Increased T2w signal indicates that there is soft tissue edema in the plantar and dorsum regions, and the plantar sinus tract is formed **(A)**. Elevated proton density weighted signal indicates that there is soft tissue edema **(B)**. Increased DWI signal in the soft tissue suggest that the water diffusion is limited **(C)**. Lower T1w signal of metatarsal bones is considered to be osteomyelitis **(D)**. A fused local APTw image and T1w image. MTRasym (3.5 ppm) is 3.8% **(E)**. Images of the foot from a DM patient without DF **(F–H)**. No abnormalities in the plantar soft tissue from the PDw image **(F)** and the T1w image **(G)**. A fused local APTw image and T1w image **(H)**. MTRasym (3.5 ppm) is 1.0%.

Fusing T1-weighted images and APTw map, ROIs on the lesions, and the surrounding muscle were manually outlined and avoided the region with extremely high or low MTRasym (at 3.5 ppm) owing to B0 or B1 inhomogeneity (shown in **Figure 2**). The location and extent of the lesions were determined by a low signal in T1-weighted images and a high signal in T2-weighted and PD-weighted images with fat suppression and DWI images.

**Figure 3:**
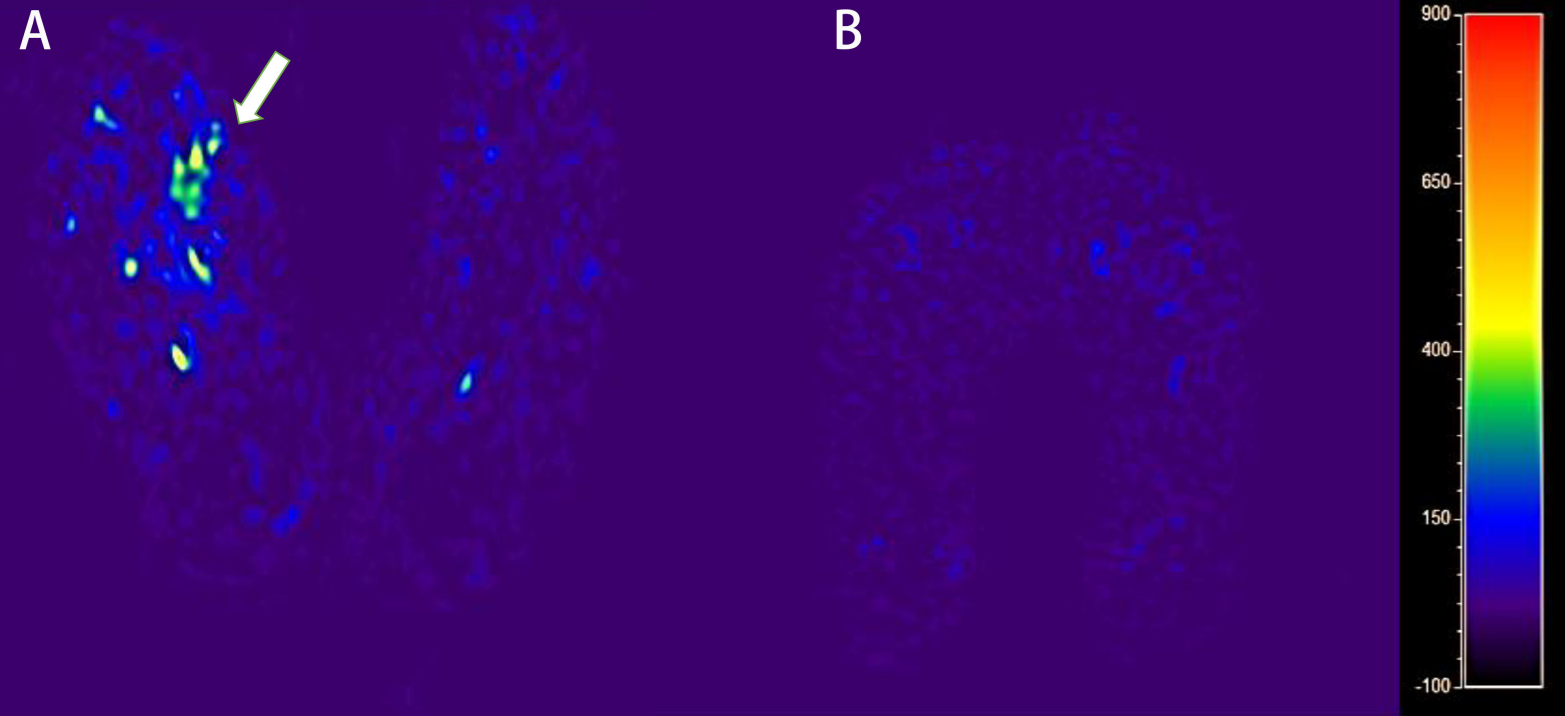
The blood flow mapping of a diabetic foot from a DM patient with DF based on the ASL image with PLD of 1,600 ms **(A)**. The blood flow mapping of a DM patient without DF based on the ASL image with PLD of 1,600 ms **(B)**.

As for ASL imaging, a circular-shaped ROI with an area of approximately 5 cm^2^ was drawn manually at the first toe metatarsal joint level of both planters on T1-weighted images fused on ASL images. For the control group, ROIs were also drawn at the plantar of the first toe metatarsophalangeal joint level and the surrounding muscle on the ASL and APTw images (**Figure 3**).

The mean of MTRasym (at 3.5 ppm) and BF in ROI was calculated on MRI console based using the formula, MTRasym (3.5 ppm) = Msat (−3.5 ppm)/M0 − Msat (+3.5 ppm)/M0, where Msat and M0 are the image signal intensities with and without radiofrequency saturation, respectively. NMTRasym (3.5 ppm) was calculated as the ratio of MTRasym (3.5 ppm) of the lesion to that of the muscle adjacent to the lesion to reduce the individual variation and B1 inhomogeneity. The rBF was calculated with the ratio of BF in the diseased area of the afflicted foot to the same area of the opposite foot.

### Statistical analysis

The software SPSS (version 17.0) was used for the statistical analysis of the data. The normal distribution of the data was detected by Shapiro–Wilk test. Logarithmic transformation was used if the data does not satisfy the normal distribution. If the data satisfied the normal distribution and with variance homogeneity, the mean ± standard deviation was used to describe the data, and the two-sample independent *t*-test was used to compare the differences between the two groups. Otherwise, the median (25th percentiles and 75th percentiles) was used to describe the data, and the non-parametric Mann–Whitney U test was used to test the difference between groups. Spearman was used to explore the relationship between nMTRasym (3.5 ppm), rBF, and blood parameters. Correlation coefficients are represented by scatter plots and linear regression. Area under the curve (AUC) was used to evaluate the diagnostic capacity of distinguishing diabetic foot (DF) from non-DF in patients with DM (18).

## Results

### Basic and clinical characteristics

The basic and clinical data of all subjects are shown in **Table 2**. There were significant differences in DM, FBG, glycosylated hemoglobin content, neutrophil percentage, and white blood cell count between the DF group and the control group (p<0.005). Meanwhile, the inter-group differences of age and gender were not statistically significant (p>0.05).

**Table 2:** The basic and clinical characteristics of the subjects in the DF and the control groups.

	DF group (n=40)	Control group (n=31)	Statistical values (t/U/χ^2^)	*p*
Age (years)	61.38 ± 13.37	59.71 ± 11.76	t = 0.548	0.585
Gender (male/female)	30/10	18/13	χ^2 = ^0.288	0.592
DM (years)	16.31 ± 7.17	10.93± 8.16	t = 2.933	**0.005**
DF (weeks)	4.30 (2.00, 12.90)	–	–	–
C-reactive protein (mg/L)	81.44 ± 78.51	8.86± 45.88	t = 4.69	**<0.001**
FBG (mmol/L)	9.28 ± 2.75	5.26 ± 0.41	t = 7.92	**<0.001**
Glycosylated hemoglobin (%)	9.80 ± 2.45	5.45 ± 0.44	t = 9.57	**<0.001**
Neutrophils ratio (%)	76.15 (61.23, 82.48)	60.80 (51.05, 71.85)	U = 166.50	**<0.001**
White blood cells (×10^9^/L)	8.73 (6.90, 12.04)	6.23 (4.52, 12.11)	U = 270.50	**<0.001**

If the data satisfied the normal distribution, mean ± standard deviation is used. Otherwise, median (25th percentiles and 75th percentiles) was used.

### The BF and nMTRasym (3.5 ppm) in the lesion of DF group

**Figure 4:**
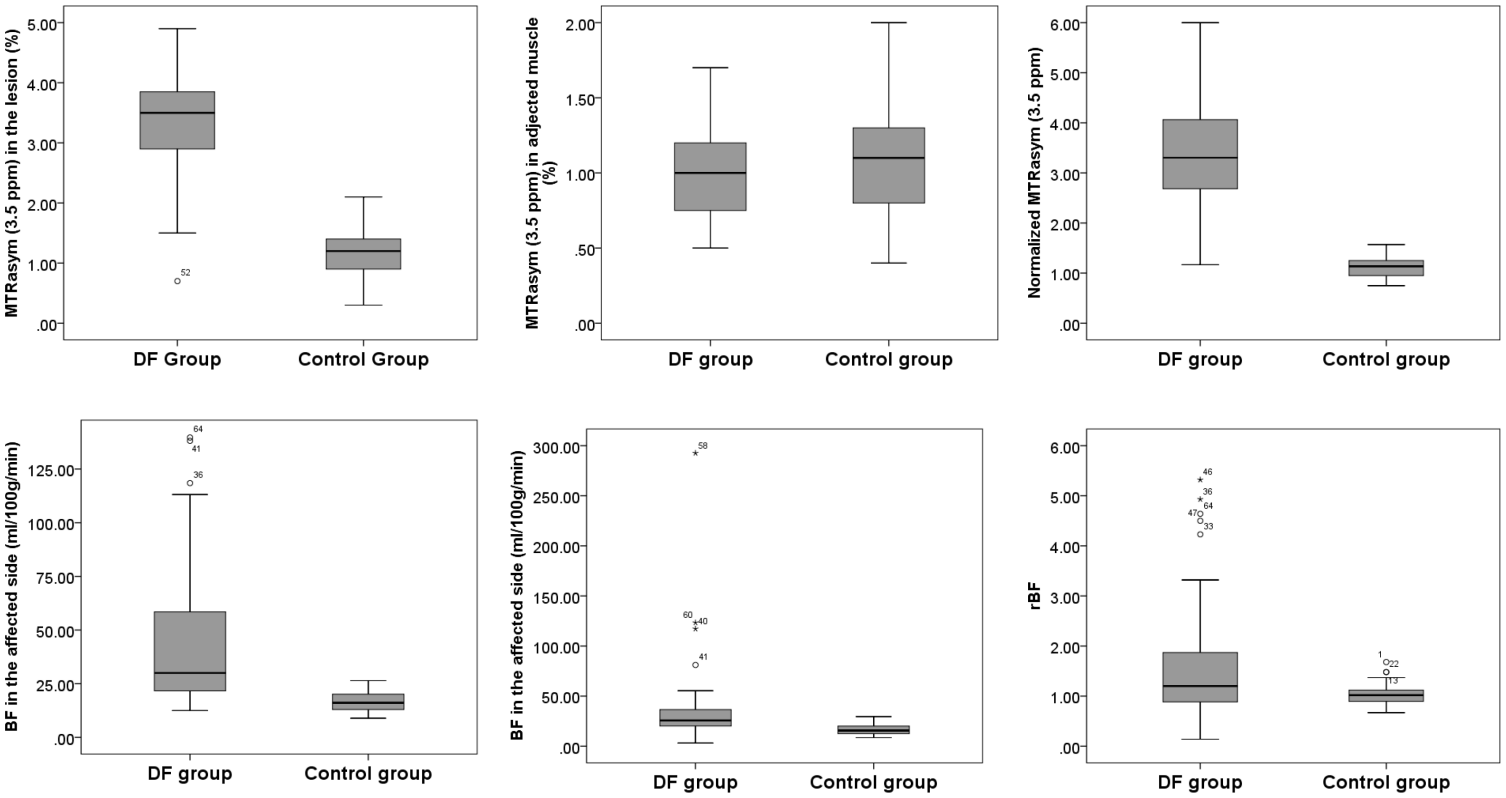
The stem and leaf plot of the foot MTRasym (3.5 ppm) in lesion, MTRasym (3.5 ppm) in adjected muscle, normalized MTRasym (3.5 ppm), BF in lesion, BF of contralateral side, and rBF in the DF group and in the control group.

Significantly higher BF in the lesion of the affected foot [30.02 (21.35, 61.34) in DF group vs. 16.10 (12.88, 20.24) mL/min/100 g in the control group, p<0.001] and in the contralateral side [24.35 (17.12, 32.98) in the DF group vs. 15.57 (12.41, 20.13) mL/min/100 g in the control group, p<0.001] was found in the DF group than in the control group. Meanwhile, elevated MTRasym (3.5 ppm) in lesion [3.30 (2.70, 3.80) in the DF group vs. 1.20 (0.8, 1.4) % in the control group, p<0.01] and nMTRasym (3.5 ppm) in lesion [3.16 (2.51, 3.98) in the DF group vs. 1.14(0.94, 1.25) % in the control group, p<0.01] in the DF group compared to that in the control group are shown **Figure 4**.

### Correlation among nMTRasym (3.5 ppm), BF, and blood biochemical parameters

**Figure 5:**
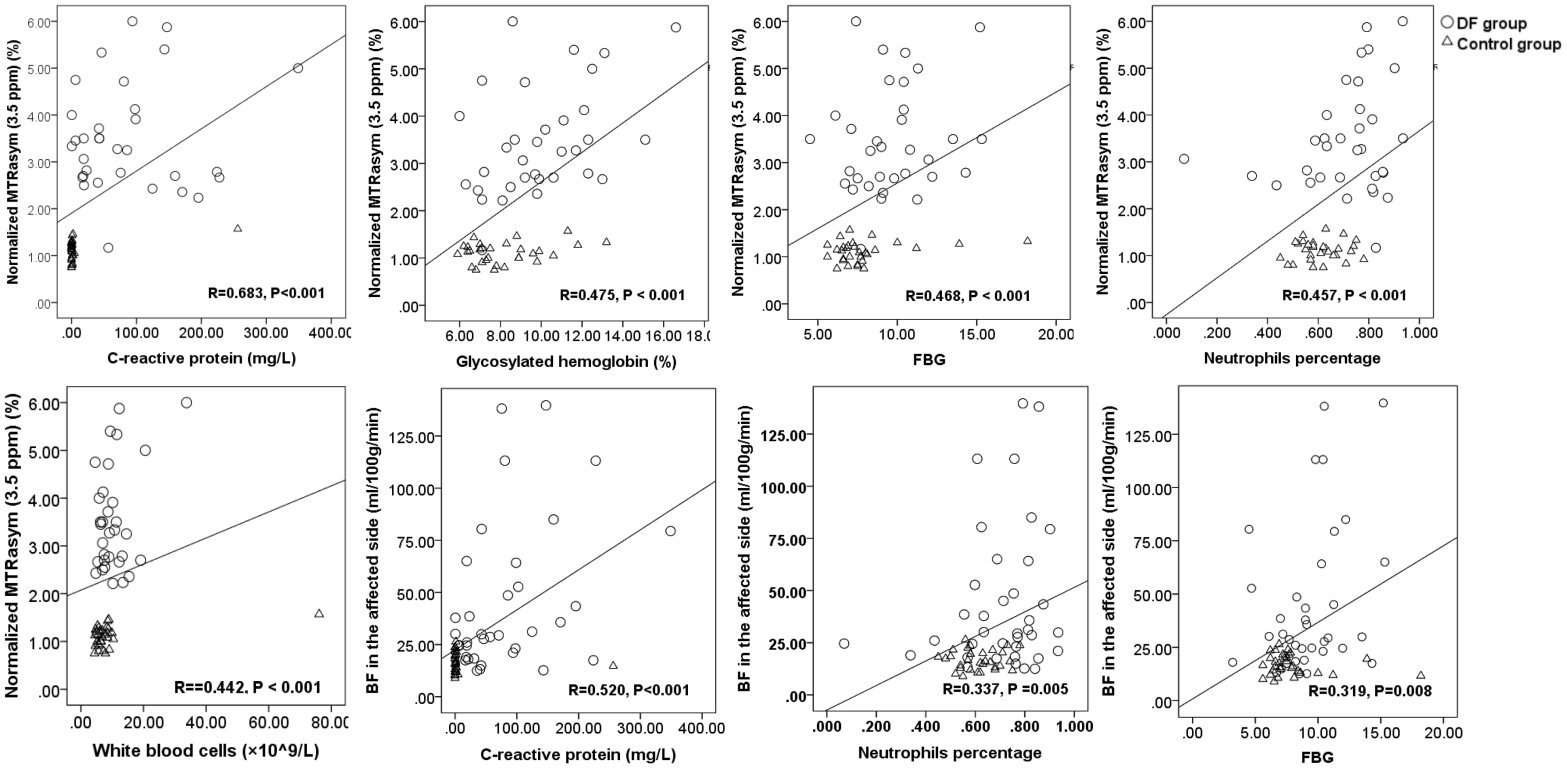
The scatter plots and linear regression of normalized MTRasym (3.5 ppm), BF, and blood biochemical parameters.

The correlations in nMTRasym (3.5 ppm) and biochemical parameters of C-reactive protein (r=0.683, p<0.001), glycosylated hemoglobin (r=0.475, p < 0.001), FBG (r=0.468, p < 0.001), neutrophil percentage (r=0.457, p < 0.001), and white blood cell (r=0.442, p < 0.001) were found. Meanwhile, a correlation between the BF in lesion and biochemical parameters of C-reactive protein (r=0.520, p<0.001), neutrophil percentage (r=0.337, p =0.005), and FBG (r=0.319, p=0.008) was observed (**Figure 5**).

### The diagnostic capacity of nMTRasym (3.5 ppm), BF, and blood biochemical parameters

**Figure 6:**
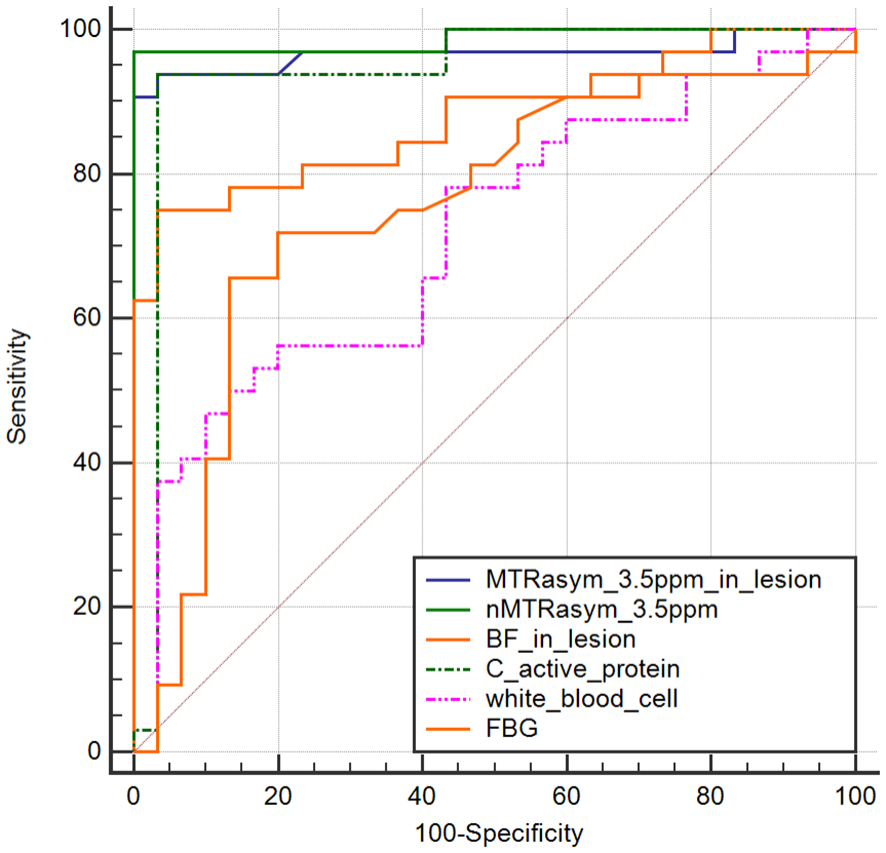
ROC curves of normalized MTRasym (3.5 ppm), BF, and blood biochemical parameters in identifying diabetic patients with/without diabetic foot.

NMTRasym (3.5 ppm) achieved AUC of 0.986 (95% CI, 0.918–1.00) in identifying with/without DF in patients with DM, which was higher than the other markers, such as MTRasym (3.5 ppm) in lesion (AUC of 0.965; 95% CI, 0.883–0.995), C-reactive protein (AUC of 0.942; 95% CI, 0.851–1.00), BF in lesion (AUC of 0.874; 95% CI, 0.764–0.945), and FBG (AUC of 0.745; 95% CI, 0.617–0.848) (**Figure 6**). The sensitivity and specificity of nMTRasym (3.5ppm) were 97.22% and 100%.

## Discussion

The application of APTw in DF was demonstrated in this study, and elevated blood flow and increased nMTRasym (3.5 ppm) in the affected foot of the DF patient were observed compared to the control group. The correlations among the nMTRasym (3.5 ppm), BF in lesion, and biochemical indicators, such as C-reactive protein, FBG, glycosylated hemoglobin, neutrophil percentage, and white blood cell, were also found, suggesting that nMTRasym (3.5 ppm) and BF in lesion could be biological markers in detecting soft tissue infection in DF. Furthermore, the AUC of local nMTRasym (3.5 ppm) is 0.986 in identifying with/without DF in patients with DM, which is higher than the C-reactive protein and BF in lesion.

To our knowledge, this is the first study to investigate diabetic foot using APTw imaging. We found elevated MTRasym (3.5 ppm) and nMTRasym (3.5 ppm) in the DF lesions in this study. In previous studies, an increased MTRasym (3.5 ppm) in the brain with intracranial infection (8) and tubercular abscess (9) had been reported, and this study is consistent with the above results. The increasing of MTRasym (3.5 ppm) may be related to the following factors. First is that the protein-rich interstitial fluid increases in the lesion of DF due to inflammation, abscess, etc. The protein content of interstitial fluid and the water with long T1 value and alkaline environment (6, 7) will directly lead to the increased MTRasym (3.5 ppm). Second, there are a large number of bacteria and microbial infections in the lesion. According to the study of bacCEST reported by Liu et al. (10), bacteria lead to the increase in the signal at MTRasym (2.6 ppm). Because of the wide range of CEST effect, it will also affect the signal at MTRasym (3.5 ppm) and lead to its indirect increase. For the same reason, the CryptoCEST contrast at 4 ppm (11) will also lead to an elevated MTRasym (3.5ppm). Third, technical factors such as non-uniform B1 field and motion during scanning (19) could also lead to abnormal increase in signal at MTRasym (3.5 ppm). However, because the MTRasym (3.5 ppm) signal in this study is also related to C-reactive protein, FBG, glycosylated hemoglobin content, and neutrophil percentage, we believe that it is mainly determined by physiological reasons instead of uniform B1 field.

The BF obtained by 3D-ASL was consistent with the BF from positron emission tomography (PET) (20). The elevated BF in the affected foot of DM patients might have been due to the increased local blood supply in the affected foot of the DM patient. In DM patients, hyperglycemia damaged the vascular intima, and the increased blood viscosity destroyed the blood vessels, leading to the insufficient blood supply to the tissues and tissue necrosis, thus aggravating the condition (20). Our results are consistent with other studies (16). Meanwhile, increased local perfusion of the affected foot was noticed after amputation in patients with diabetic foot ulcers (1). Impaired microvascular permeability in tibial anterior muscles and larger T1-weighted signal characteristics of osteomyelitis were observed by DCE-MRI (10, 11).

This study has some limitations. First, radio frequency field (B1) inhomogeneous on the APTw images and the effect of tissue T1 fail to be corrected due that fact that B1 and T1 mapping has not been collected, although the magnetic field (B0) inhomogeneity of MTRasym (3.5 ppm) was corrected. In the future study, B1 mapping and T1 mapping will be acquired to improving the interpretability of the results. Second, manual delineation of ROI is the main technique in this study, and future study will be based on methods such as image segmentation and registration to achieve automatic drawing of lesions or particular muscles to increase the objectivity of the results. Third, as the sample size increases, we will make groupings by the pathological level to further investigate the value of nMTRasym (3.5 ppm) and rBF to evaluate the infection of DF.

In summary, DF infection was assessed using both of APTw and ASL imaging for the first time, and the elevated nMTRasym (3.5 ppm) and BF in lesion may be used as novel indicators in evaluating the DF in DM patients with safer and more convenient treatment for patients with renal insufficiency due to its non-contrast injection.

## Data availability statement

The raw data supporting the conclusions of this article will be made available by the authors, without undue reservation.

## Ethics statement

The studies involving humans were approved by Chu Hsien-I Memorial Hospital and Tianjin Institute of Endocrinology (approval number: DXBYYkMEC2021-09). The studies were conducted in accordance with the local legislation and institutional requirements. The participants provided their written informed consent to participate in this study.

## Author contributions

SL: Supervision, Validation, Writing – review & editing. JT: Writing – review & editing. SZ: Writing – original draft. XS: Writing – review & editing. XM: Writing – original draft. GM: Writing – original draft. DL: Writing – original draft. ZS: Writing – review & editing. BC: Writing – review & editing.
